# The Vγ4/butyrophilin conspiracy: novel role of intraepithelial γδ T cells in chronic inflammatory bowel disease

**DOI:** 10.1038/s41392-023-01697-w

**Published:** 2023-11-18

**Authors:** Dieter Kabelitz

**Affiliations:** Institute of Immunology, Christian-Albrechts University Kiel and University Hospital Schleswig-Holstein Campus Kiel, Kiel, Germany

**Keywords:** Medical research, Biomarkers

In a recent article published in *Science*, Dart and colleagues reported that colonic T cells expressing the Vγ4 T-cell receptor (TCR) interact with epithelial butyrophilin-like (BTNL) proteins BTNL3 and BTNL8 in healthy individuals, and they observed a depletion of Vγ4 T cells in inflammatory bowel diseases with renormalization associated with disease remission.^1^ This paper identifies the BTNL/Vγ4 axis as a new disease modifier in IBD.^1^

Conventional T cells express the αβ TCR and recognize peptides presented by MHC class I (CD8) or MHC class II (CD4) molecules. In contrast, γδ T cells are MHC-independent and fulfill important functions in immunosurveillance against stressed and infected cells. Moreover, studies (so far mainly in the murine system) also indicate a crucial role of γδ T cells in monitoring tissue integrity. The best-studied subset of human γδ T cells expresses a Vγ9Vδ2-encoded TCR. These cells account for the majority of peripheral blood γδ T cells but are less frequent in tissues. Vγ9Vδ2 T cells recognize pyrophosphate molecules (“phosphoantigens”) in a strictly butyrophilin (BTN) 3A1 and 2A1-dependent manner.^2^ BTN and BTN-like molecules (BTNL) are Ig superfamily-member, B7-related transmembrane proteins with multiple roles in the immune system and beyond. Phosphoantigens are overproduced in cancer cells due to a dysregulated mevalonate pathway (isopentenyl pyrophosphate, IPP), or accumulate in infected cells upon infection with microbes producing (*E*)-4-Hydroxy-3-methyl-but-2-enyl pyrophosphate (HMBPP). Such phosphoantigens bind to intracellular regions of BTN3A1 and induce a conformational rearrangement of BTN2A1/3A1 complex which is then recognized by the Vγ9Vδ2 TCR (Fig. 1a). As a consequence, Vγ9Vδ2 T cells can kill a variety of cancer cells because they sense the increased levels of IPP, but they do not depend on recognition of tumor-specific or tumor-associated antigens. Together with the fact that γδ T cells are not HLA-restricted, these features have raised a recent burst of interest to bring γδ T cells into clinical application in cancer immunotherapy.^3^

**Fig. 1:**
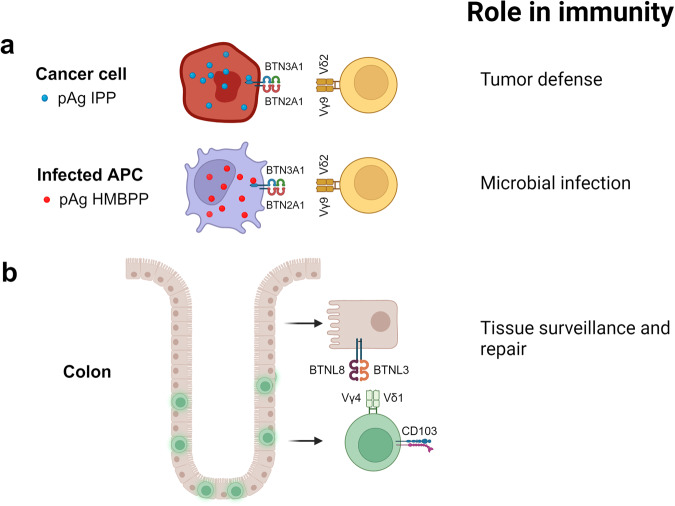
Differential role of human γδ T-cell subsets in immune surveillance. **a** Vγ9Vδ2 T cells are the dominant subset of γδ T cells in peripheral blood. They recognize phosphoantigens (pAg) overproduced in transformed cells (isopentenyl pyrophosphate, IPP) or accumulating in infected cells ((*E*)-4-Hydroxy-3-methyl-but-2-enyl pyrophosphate, HMBPP) in a strictly BTN3A1/2A1-dependent manner. Vγ9Vδ2 T cells play important roles in tumor defense and control of microbial infection. **b** Intestinal intraepithelial Vγ4Vδ1 T cells can directly interact with BTNL3/BTNL8 heterodimers expressed on epithelial cells. A subset of intestinal Vγ4 T cells expresses CD103 (αEβ7 integrin), a marker of IEL and T_RM_ T cells. CD103 is a receptor for E-cadherin on endothelial cells. These cells are important for tissue surveillance and repair and are compromised in IBD. Figure created with BioRender.com

Distinct functions have been identified for tissue-resident epithelial γδ T cells in the skin and intestine where they contribute to barrier surveillance but also to tissue integrity and repair. Elegant mouse models have discovered the essential role of specific BTNL proteins expressed in a tissue-selective manner for the selection of specific γδ TCR repertoires. For instance, *Skint1* and *Skint2* (BTNL-related molecules) are required for the selection of dendritic epidermal Vγ5 γδ T cells, while *Btnl6* and *Btnl1* are required for the development of intestinal Vγ7 γδ T cells.^4^ The intra-epithelial γδ T cells contribute by multiple pathways to local tissue repair and immune defense, for instance by the production of soluble mediators like IGF1, KGF, IFNγ, GZMA/B, and others.

While models relying on genetic deficiency of a given *Btnl* gene have clearly demonstrated the importance of tissue-specific selection of γδ T cells and their importance for tissue homeostasis and immune surveillance in mice, similar studies in humans are naturally much more demanding. In a previous study from the Hayday group, it was already shown that the human colon harbors intraepithelial Vγ4-expressing γδ T cells which can specifically interact with BTNL3/BTNL8 heterodimers expressed on the colonic epithelial cells (Fig. 1b).^5^ However, it remained unresolved whether the reciprocal liaison of intraepithelial Vγ4γδ T cells with epithelial BTNL3/BTNL8 ligands has any implications for chronic inflammatory bowel diseases (IBD) like Crohn’s disease (CD) and ulcerative colitis (UC). Both diseases are growing in incidence and are essentially incurable, which raises the importance of new insights into new pathways that might be druggable.

In their recent paper, Dart and colleagues performed an unprecedented in-depth study to characterize the colonic γδ TCR repertoire in healthy donors and IBD patients, and they investigated the impact of BTNL3 and BTNL8 on the selection of Vγ4 γδ intraepithelial cells (IEL).^1^ These studies were performed on a total of 173 colonic endoscopic biopsies including non-IBD controls, uninflamed gut from patients with confirmed IBD, and inflamed lesions from IBD patients. First, they identified among Vδ2-negative γδ IEL a major subset (around 47%) that expressed Vγ4 and the integrin CD103 as a marker of IEL and tissue-resident memory (T_RM_) T cells. CD103 is a receptor for E-cadherin on epithelial cells. The transcriptomic analysis of CD103^+^Vγ4 T cells revealed an enrichment of genes important for effector function, activation and migration (e.g., *KIR2DLA, TIGIT, CCR9, GZMA, FGF9*) when compared to CD103^neg^Vγ4 IEL. They used HEK392T cells transduced with *BTNL3* + *BTNL8* to interrogate the activation of the CD103^+^Vγ4 IEL. They observed a specific TCR down-regulation together with up-regulation of 41BB and GZMB but little if any cytokines like TNFα, IFNγ, and no mobilization of CD107 as a surrogate marker of cytotoxic activity. Importantly, these cells were also largely refractory to activation with phorbolester plus ionomycin, and thus displayed a selective pattern of responsiveness. Further characterization of the CD103^+^Vγ4 IEL revealed high expression of FcεRIγ (involved in signal transduction) and NK receptors NKp46 and NKG2C. These studies have thus far characterized in detail the phenotype and activation of Vγ4 IEL in the healthy human colon.

Next, Dart and colleagues expanded their study to IBD patients. Overall, they observed a significantly lower γδ T-cell frequency in inflamed as opposed to healthy gut. More detailed analysis revealed a disproportionate loss of CD103^+^Vγ4 cells. To investigate if the decline of intestinal γδ T cells in IBD might be due to antigen-driven activation and subsequent deletion, they performed Vγ TCR repertoire analysis. Interestingly, this revealed increasing diversity in IBD *versus* control, indicating that the loss of Vγ4 IEL was not driven by antigenic pressure.

To obtain genetic evidence for the role of BTNL3 + BTNL8 for the selection of Vγ4 cells in humans is difficult. The authors had the opportunity to analyze healthy donors homozygous for a BTNL8*3 copy number variation in which deletion causes an in-frame fusion of the BTNL8 N terminus to the BTNL3 C terminus resulting in inefficient cell surface expression. These individuals had strongly reduced numbers of colonic CD103^+^Vγ4 IEL, which elegantly supports the notion that BTNL3 and BTNL8 are indeed required for the selection of human Vγ4 IEL. In line, they found in a large CD cohort a significant association between BTNL3 loss of function and penetrating (B3) disease.

Finally, they addressed the question how the Vγ4 IEL compartment can be modulated in IBD. Vγ4 IEL cells express RNA encoding receptors for inflammatory cytokines, and the authors found that culturing primary gut T cells in IL12 + IL18 changed the phenotype of Vγ4 cells with significant reduction of CD103, TIGIT, NKp44, suggesting that the inflammatory cytokine milieu might have similar effects in vivo. Vice versa, they observed in macroscopically healed mucosa from several UC patients an increased proportion of CD103^+^Vγ4 IEL, indicating that these cells might recover locally upon effective treatment. Moreover, their preliminary investigation with 19 IBD patients suggests that reconstitution of colonic CD103^+^Vγ4 IEL might be associated with sustained remission. As the authors discuss, monitoring of specific immune subsets like CD103^+^Vγ4 cells (which can be performed upon routine endoscopic biopsy) might be a useful novel biomarker to assess disease activity in IBD.

Taken together, this study has uncovered novel insights into the intricate role of a specific human γδ T-cell subset in intestinal immune surveillance. The selective depletion of CD103^+^Vγ4 cells in IBD, linked with deficiencies in BTNL3/BTNL8 expression, and the possible reconstitution of the γδ IEL compartment upon remission reported in this paper^1^ paves the way for similar studies in other barrier diseases affecting, e.g., the lung or skin.

## References

[1] Dart RJ (2023). Conserved γδ T cell selection by BTNL proteins limits progression of human inflammatory bowel disease. Science.

[2] Rigau M (2020). Butyrophilin 2A1 is essential for phosphoantigen reactivity by γδ T cells. Science.

[3] Kabelitz D, Serrano R, Kouakanou L, Peters C, Kalyan S (2020). Cancer immunotherapy with γδ T cells: many paths ahead of us. Cell. Mol. Immunol..

[4] Jandke A (2020). Butyrophilin-like proteins display combinatorial diversity in selecting and maintaining signature intraepithelial γδ T cell compartments. Nat. Commun..

[5] Melandri D (2018). The γδTCR combines innate immunity with adaptive immunity by utilizing spatially distinct regions for agonist selection and antigen responsiveness. Nat. Immunol..

